# The Decreased *Treg* Cells Number Associated with Retinal Lesion Size in Ocular Toxoplasmosis

**DOI:** 10.1155/2024/3495376

**Published:** 2024-01-24

**Authors:** Ovi Sofia, Muna Amalia, Herryanto Thomassawa, Loeki Enggar Fitri, Seskoati Prayitnaningsih, Hani Susianti

**Affiliations:** ^1^Doctoral Program in Medical Science, Faculty of Medicine, Universitas Brawijaya, Malang, Indonesia; ^2^Department of Ophthalmology, Faculty of Medicine, Universitas Brawijaya, Dr. Saiful Anwar General Hospital, Malang, Indonesia; ^3^Residency Training Program, Department of Ophthalmology, Faculty of Medicine, Universitas Brawijaya, Dr. Saiful Anwar General Hospital, Malang, Indonesia; ^4^Department of Clinical Parasitology, Faculty of Medicine, Universitas Brawijaya, Dr. Saiful Anwar General Hospital, Malang, Indonesia; ^5^Department of Clinical Pathology, Faculty of Medicine, Universitas Brawijaya, Dr. Saiful Anwar General Hospital, Malang, Indonesia

## Abstract

**Introduction:**

The imbalance of the immune response is an important factor contributing to the incidence of ocular toxoplasmosis (OT). Regulatory T cells (Treg) play a key role in maintaining the balance between Th1 and Th17 immune responses, while interleukin-27 (IL-27) levels are related to the differentiation of Th17 cells. This study analyzes the differences in the number of Treg cells and the level of IL-27 between OT patients and seropositive individuals without ocular lesions and its correlation with retinal lesion size.

**Methods:**

This analytic observational study, conducted for 8 months, involved 11 OT patients and 10 seropositive individuals without ocular lesions. All subjects underwent a comprehensive ophthalmological examination. Retinal lesions were documented by fundus photographs and the size was measured using Digimizer 4.2.2.0 software. Isolation of peripheral blood mononuclear cells (PBMC) was performed to measure the number of Treg cells using flow cytometry and interleukin-27 levels were assessed using the Sandwich enzyme-linked immunosorbent assay (ELISA) technique. Data were analyzed with SPSS.

**Result:**

The number of Treg cells in the OT group (47.16 ± 15.66%) was lower than in the seropositive group without the ocular lesions (62.86 ± 17.08%) (*p* = 0.029). The serum IL-27 levels in the OT group were not significantly different from the seropositive group without the ocular lesions (*p* = 0.360). The number of Treg cells was significantly related to retinal lesion size (*p* = 0.043), with a correlation coefficient of −0.648, indicating a strong and inverse correlation. There was no significant correlation between serum IL-27 levels and retinal lesion size (*p* = 0.556).

**Conclusion:**

Ocular toxoplasmosis patients have a low number of Treg cells that are inversely related to the retinal lesion size. The size of the retinal lesion increases as the number of Treg cells decreases.

## 1. Introduction

Ocular toxoplasmosis (OT) is caused by *Toxoplasma gondii* (*T. gondii*) infection in the eye and is the most common cause of posterior uveitis worldwide. It is estimated that one-third of the world's population has been infected with *T. gondii* but with different manifestations. A study in the United States stated that 2% of people with seropositive could manifest ocular toxoplasmosis [1–4]. In Indonesia, Aditama et al. mentioned that the prevalence of toxoplasmosis is about 2%–80%, which varies in each region. The study by Sofia and Hariyono at Dr. Saiful Anwar General Hospital Malang for three years found 48 eyes (38 patients) with retinal lesions due to *T. gondii* infection [5, 6].

The high prevalence of seropositivity, but only the small percentage that leads to ocular manifestations, raises the question of what factors cause the appearance of clinical manifestations in the eyes of individuals who are both infected with *T. gondii*. Three factors play a major role in clinical manifestations due to *T. gondii* infection: host immunity, parasite inoculum, and strains or genotypes of *T. gondii* [7, 8].

The exact mechanism of how *T. gondii* infection can reach the eye and cause clinical manifestations in patients is still being studied. The immune response as an essential factor in *T. gondii* infection is thought to be closely related to the role of cytokines. Various studies have found that Th17 cells play a role in eye tissue damage due to *T. gondii* infection. It is also mentioned that IL-27 is a potent inhibitor in Th17 cell differentiation. Regulatory T cells (*Treg*) are associated with the Th17 immune response, which plays a key role in maintaining the balance of immune response to maintain immune homeostasis. *Treg* cells are a subpopulation of T cells that function to suppress the excessive response of B cells and T cells to other antigens so that the immune response does not continue after it is unnecessary [9–13].

Several studies have been conducted related to OT and its correlation with immune responses, but the research in humans is still limited [14–16]. Most studies have been conducted in rats, and current human studies are limited to aqueous humor samples, which collection process is invasive. This study evaluates the role of *Treg* cells and IL-27 in ocular toxoplasmosis and their correlation with the retinal lesion size, using blood serum samples from OT patients.

The retinal lesion size is selected as the main parameter as it is one of the clinical manifestations of OT. The lesions on the retina can be active, i.e., yellow-colored focal lesions or hyperpigmented retinochoroidal scars in inactive cases.

## 2. Methods

This study is an analytic observational with a cross-sectional approach which was conducted at the Ophthalmology Outpatient Clinic and Clinical Pathology Laboratory of Dr. Saiful Anwar General Hospital Malang, as a tertiary referral hospital, and Biomedical Laboratory of the Faculty of Medicine, Universitas Brawijaya, for 8 months during 2022-2023. This study has been approved by the Health Research Ethics Commission of Dr. Saiful Anwar General Hospital Malang with number 400/155/K.3/102.7/2022.

We used a consecutive sampling method, which recruited all patients with OT and seropositive individuals without ocular lesions who met the inclusion criteria. The inclusion criteria were aged 10–60 years, immunocompetent, agreed to participate, and signed the informed consent. Meanwhile, individuals with reactive VDRL/TPHA, those unable to complete all examinations, or those with damaged blood samples were excluded. The seropositive subjects without ocular lesions were recruited from healthy volunteers. The pregnancy test was performed in female subjects of childbearing age. All of the subjects agreed to participate in this study.

### 2.1. Ophthalmological Examination

Primary data collection was carried out by history taking from all subjects. In female subjects of childbearing age, a pregnancy test is carried out; if it is negative, the subject is recruited. A complete ophthalmological examination was then performed. The anterior segment was examined using a slit-lamp biomicroscope and the posterior segment was evaluated using a binocular indirect ophthalmoscope and fundus photograph.

### 2.2. Serological Examination

The blood samples were taken and divided into two parts. A blood sample in a serum separator tube (SST) was used to examine IgG and IgM anti-*Toxoplasma*. A serological test was performed to confirm the exposure of *T. gondii*.

### 2.3. Interleukin-27 Levels Measurement

Interleukin-27 levels were measured using the Sandwich ELISA method (ELISA KIT E-EL-H2338 by Elabscience).

### 2.4. *Treg* Cells Count

#### 2.4.1. The Isolation of Peripheral Blood Mononuclear Cells (PBMC)

Blood samples with EDTA were immediately processed to isolate unstimulated PBMC. A 15 ml centrifuge tube was prepared and filled with Ficoll–Hypaque *d* = 1.077 g/ml (ratio 1 : 1) with the number of blood samples and then reversed slowly to make a homogeneous solution, mixed 1 : 1 with *Phosphate Buffer Saline* (PBS), and centrifuged at room temperature at a speed of 1600 rpm for 30 minutes. After centrifugation, the PBMC ring was slowly taken using a micropipette and placed in a new 15 ml centrifuge tube.

The PBMC solution was washed with 10 ml PBS and centrifuged at room temperature at a speed of 1200 rpm for 10 minutes. The supernatant was discarded and the cell pellets formed were washed with the PBS, at a room temperature of 1200 rpm for ten minutes, repeated twice. After centrifugation, the pellets (PBMC cells) are formed at the base of the tube. The pellets are dissolved with PBS 1 ml in an Eppendorf 1.5 ml tube and stored in a refrigerator at a temperature of −40°C.

#### 2.4.2. Flow Cytometry Procedure

In this study, we used separate antibodies, including PE antihuman FOXP3 antibody, PE/Cy5 antihuman CD25 antibody, FITC antihuman CD4 antibody, and also FOXP3 Fix/Perm Buffer Set by BioLegend. We used BD FACSCALIBUR 1 laser and 3 color machine.

The PBMC cell pellets were stained with cell surface antibodies (CD4+ and CD25+) first and then with intracellular (nuclear) markers for FCM (*Treg*).

Cell pellets are washed with the flow cytometry staining buffer and then centrifuged at 2500 rpm for 3 minutes at 4°C. The supernatant is removed and the cell pellets formed are ready to be stained with the cell surface antibody. First, mix the antihuman CD4 FITC-conjugated and antihuman CD25 PE/Cy.5-conjugated with the flow cytometry staining buffer. A 50 *μ*l solution is mixed with cell pellets and then incubated at room temperature for 20 minutes in the dark. After that, 300 *μ*l of flow cytometry staining buffer was added and rehomogenized for continued intracellular (nuclear) staining.

The suspension was centrifuged at 2500 rpm for 3 minutes, at 4°C. The cell pellets formed are washed by adding 500 *μ*l of 1x FoxP3 fixation/permeabilization buffer solution and then centrifuged like before. The cell pellets formed are washed by adding 500 *μ*l of 1x FoxP3 fixation/permeabilization buffer solution, homogenized, and then incubated at room temperature for 20 minutes in the dark. After incubation, they are centrifuged like before. Then, they repeated the procedure, without incubating in the dark room. The cell pellets were mixed with 500 *μ*l of 1x FoxP3 permeabilization buffer solution and then incubated at a room temperature for 20 minutes in the dark. The cell pellets are ready to be stained with the intracellular nuclear antibody. Then, antihuman FoxP3 PE-conjugated antibody with 1x FoxP3 permeabilization buffer was mixed. A 50 *μ*l solution was mixed with cell pellets and then incubated at room temperature, for 20 minutes, in the dark. 500 *μ*l of 1x FoxP3 permeabilization buffer solution was added and then centrifuged at 2500 rpm for 3 minutes, at 4°C. The cell pellets were mixed with 300 *μ*l flow cytometry staining buffer and then transferred to the FCM cuvette and ready to read in flow cytometry device.

### 2.5. The Retinal Lesion Size Measurement

**Figure 1:**
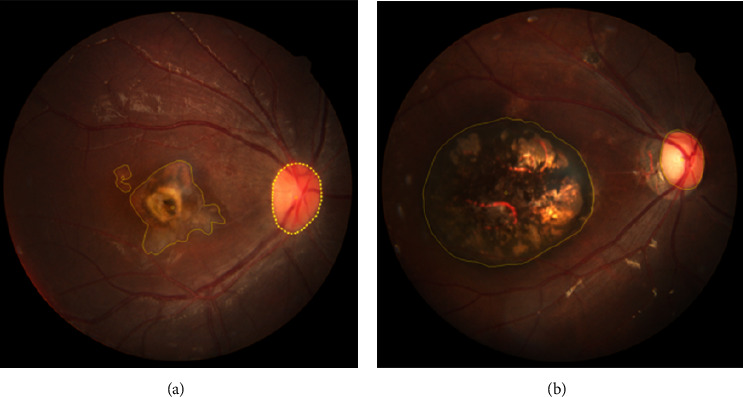
Retinal lesion measurement using Digimizer 4.2.2.0 in (a) an active recurrent lesion and (b) an inactive lesion with hyperpigmented retinochoroidal scar.

The retinal lesion size in the OT group was examined using fundus photographs and measured with the Digimizer 4.2.2.0 software, performed in disk-diameter (dd), as shown in Figure 1. In patients with bilateral retinal lesions, measurements are taken in the active lesion or the larger lesion if both lesions are active or inactive. The results of the retinal lesion measurements were then confirmed by an infection and immunology ophthalmologist consultant.

## 3. Result

### 3.1. Characteristics of the Subjects

There were 21 subjects consisting of 11 subjects in the OT group and 10 subjects in the seropositive group without ocular lesions. The characteristics of the subjects are presented in Table 1, most of the subjects were 19–45 years of age in both groups (71.43%), in the OT group (45.45%) and in the seropositive group without ocular lesions (90%). Based on the sex predominance, most of the subjects are women (71.43%), either in the OT group (63.64%) or the seropositive group without ocular lesions (80%). Based on the result of serological tests of anti-*Toxoplasma* antibodies, 100% had positive IgG titers, while 23.81% had positive IgM titers.

The ophthalmological conditions in the OT group are presented in Table 2. It was found that 63.64% of OT patients had unilateral lesions, and most of the lesions were active lesions (63.64%). Most of the active OTs were recurrent cases (85.71%). Ten patients (90.9%) had macular lesions.

### 3.2. Comparison between the Number of *Treg* Cells in OT and Seropositive Groups without Ocular Lesions

There was no significant difference between the number of *Treg* cells in the active OT and inactive OT (*p* = 0.098) (Table 3). So, we further analyzed the difference in the number of *Treg* cells between the OT group and seropositive individuals without ocular lesions.

**Figure 2:**
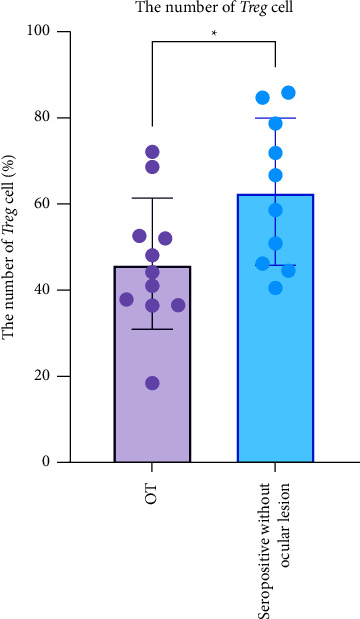
The number of *Treg* cells in the OT and seropositive groups without ocular lesions. The average number of *Treg* cells in the OT group was lower than the seropositive group without ocular lesions.

**Figure 3:**
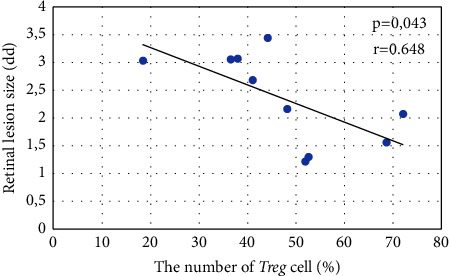
The correlation between the number of *Treg* cells and the retinal lesion size. The greater the number of *Treg* cells, the smaller the retinal lesion size.

The number of *Treg* cells in the OT group was significantly lower than the number of *Treg* cells in the seropositive group without ocular lesions (*p*=0.029) (Figure 2). Measurement of the number of *Treg* cells in the OT group showed a significant correlation with the retinal lesion size (*p*=0.043) (Figure 3).

### 3.3. Comparison between the IL-27 Levels in OT and Seropositive Groups without Ocular Lesions

**Figure 4:**
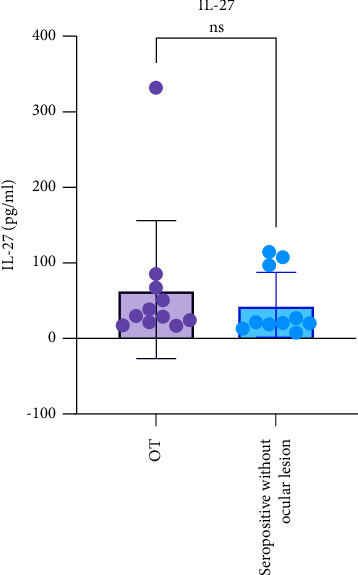
The IL-27 levels in the OT and seropositive groups without ocular lesions showing no significant differences between the two groups.

This study found no significant difference between serum IL-27 levels in the OT and seropositive groups without ocular lesions (*p*=0.360). Serum IL-27 levels also did not significantly correlate with retinal lesion size in OT (*p*=0.556) (Figure 4).

## 4. Discussion

### 4.1. Characteristics of Subjects

Most of the study subjects (71.43%) were adults (19–45 years). This finding was also found in the OT group, which was 45.45% and the seropositive group was 90%. This finding is in accordance with the previous study by Cifuentes-González et al. in an epidemiological study of OT in California (2015–2019), where 65.2% of subjects were in the 15–49 year age group [17]. Bustillo et al. also found that the highest incidence and prevalence of active OT each year is at 25–44 years old [18]. Gilbert et al. 1999; Bosch-Drissen et al. 2002, also reported that patients aged 25–45 years were the largest group suffering from OT, 78–82% [19, 20].

We also found that most of the subjects were women, with 63.64% in the OT group and 80% in the seropositive group without ocular lesions. Tabatabaei et al. reported the highest prevalence of OT in women, 60% (*n* = 24); Sofia and Hariyono reported that 66% (*n* = 25) of OT cases occurred in women; and Mendes et al. said that the highest proportion of seropositive toxoplasmosis were in women, 64.6% (*n* = 75) [6, 17, 21, 22]. Several studies reported no significant difference between males and females in the prevalence of OT [20, 23, 24]. Ferreira et al. reported a total of 349 patients, 53.6% (*n* = 187) were male, and 46.4% (*n* = 162) were female [23]. The high prevalence of toxoplasmosis in women especially in childbearing age in this study may be because women have higher consideration to do anti-*Toxoplasma* antibody testing for screening purposes before planning a pregnancy.

### 4.2. Comparison between the Number of *Treg* Cells in OT and Seropositive Groups without Ocular Lesions

This study found that the number of *Treg* cells in the OT group was lower than that in the seropositive group without ocular lesions. This is in line with the results of a study by Oldenhove et al. [14, 25]. A lower number of *Treg* cells may play an important role in the infection process. In the OT group, the number of *Treg* cells was less, so it may contribute to clinical manifestations. A study on *Treg* cells in another eye infection entity, ocular tuberculosis, by Basu et al. also found that the number and function of *Treg* cells were decreased [26].

A study that analyzed the correlation between the number of *Treg* cells and the retinal lesion size has not been reported. This study found a significant correlation between the number of *Treg* cells and the retinal lesions size with a strong and negative correlation coefficient, indicating an inverse relationship between the number of *Treg* cells and the retinal lesions size. In previous research by Cordeiro et al. that analyzed the correlation between IL-10 cytokine and retinal lesion size, it was found that there was a negative correlation between retinal lesion size and IL-10 [27].

Increased number of *Treg* cells will increase the production of IL-10, decreasing Th1 and IFN-*γ* levels. An increased number of *Treg* cells will activate TGF-*β*, reducing the level of proinflammatory IL-17, so that retinal damage can be minimized. Meanwhile, if the number of *Treg* cells decreases, TGF-*β* and IL-10 will also decrease, while IL-17 and Th1 will increase. Increased IL-17, which is a proinflammatory cytokine, will facilitate the occurrence of retinal damage.

### 4.3. Comparison between IL-27 Levels in OT and Seropositive Groups without Ocular Lesions

This study showed that there was no difference between serum IL-27 levels in the OT group and seropositive group without ocular lesions. In a study by Maia et al., an analysis of the gene encoding IL-27 via PBMC mRNA was carried out between OT patients and seropositive without ocular lesions; it was found that there was no difference in IL-27 mRNA levels in the OT group and seropositive group without ocular lesions [28]. Research by Tong et al. reported that there was a significant increase in the mRNA level of IL-27p28 starting on day 2 in ocular tissue and starting on day 6 in the cervical lymph tissue of the mice model infected with *T. gondii* compared to control mice. These results indicate that IL-27/IL-27R induced by *T. gondii* infection plays a role in the immunopathology of OT [29].

Various studies have reported that IL-27 is pleiotropic in some T helper subsets. The first time IL-27 was discovered, its role was reported as a cytokine that induces Th1 cells that are protective in infection processes. Several subsequent studies reported the effect of IL-27 as a potent inhibitor of Th17 differentiation in autoimmune encephalitis and cerebral toxoplasmosis [30]. The nature of IL-27 may explain the result of this study, which found no difference between serum IL-27 levels in the OT group and the seropositive group without ocular lesions.

The mechanism underlying the occurrence of retinal damage in *T. gondii* infection is not fully understood, the immune response is thought to play a direct role in the pathogenesis of retinochoroiditis, and cytokines play a fundamental part. Th17 cells, essential mediators in inflammatory and autoimmune reactions in uveitis, are thought to play a significant role in the host's immune response to retinal tissue damage due to *T. gondii* infection [30, 31].

A study by Carneior et al., which evaluated the effect of cytokines on OT, showed that in infants with OT, there was an increase in IL-1*β* and TNF-*α* levels, babies with active lesions of retinochoroiditis had increased levels of IFN-*γ* and IL-17, infants with retinochoroidal scars have increased IL-10, IL-1*β*, and TNF-*α*, infants with active lesions and retinochoroidal scars have increased IL-10, and in general, there are increased levels of IFN-*γ* and IL-12 in all clinical conditions [16].

Another study by Sauer et al. showed that in rats with OT, there was an increase in IL-17 levels in the primary infection, but these IL-17 levels then decreased, followed by an increase in IL-27 levels in recurrence cases. It was concluded that IL-27 is the main cytokine that plays a role in inhibiting the Th17 immune response. IL-27 also plays a role in activating the Th1 immune response, which plays an important role in the eradication process of parasites [15].

In this study, there was no correlation between serum IL-27 levels and the retinal lesion size in OT. According to the researchers, these findings indicated that the process of retinal tissue damage is not directly influenced by IL-27 activity but involves various other inflammatory mediators that may be initiated by Th17 cell activation. Other factors, such as *T. gondii* strains and genetic polymorphisms, may influence the host's immune response to *T. gondii* infection [31–33].

## 5. Conclusion

This study showed that the number of *Treg* cells in the OT group was lower than in the seropositive group without ocular lesions and the number of *Treg* cells was inversely related to the retinal lesion size in OT. Meanwhile, there was no difference in serum IL-27 levels between the OT and seropositive groups without ocular lesions.

## Figures and Tables

**Table 1:** Characteristics of the subjects.

Variable	OT		Seropositive without ocular lesion		Total		Mean ± SD	*p* value
	*n*	%	*n*	%	*n*	%		
Age (years)								
10–18	4	36.36	0	0	4	19.05	29.24 ± 6.44	0.102
19–45	6	45.45	9	90	15	71.43		
≥46	1	9.09	1	10	2	9.52		
Sex								
Male	4	36.36	2	20	6	28.57		0.635
Female	7	63.64	8	80	15	71.43		
Anti-*Toxoplasma gondii* antibodies (+)								
IgG	9	81.82	7	70	16	76.19		
IgG and IgM	2	18.18	3	30	5	23.81		

**Table 2:** Ophthalmological conditions of the OT group.

	Frequency	%
Laterality		
Unilateral	7	63.64
Bilateral	4	36.36
Lesion activity		
Active lesion	7	63.64
Primary	1	14.28
Recurrent	6	85.71
Number of scar(s)		
1 scar	3	50
2 scars	1	16.6
3 scars	1	16.6
5 scars	1	16.6
Retinochoroidal scar	4	36.36
Lesion location		
Macula	10	90.9
Extramacula	1	9.09

**Table 3:** The number of *Treg* cells.

Group	Mean ± SD (%)	*p* value
Active OT	46.3457 ± 11.60736	0.098
Inactive OT	45.8950 ± 22.39106	
Seropositive without ocular lesion	62.8590 ± 17.07842	

## Data Availability

The dataset used to support the findings of this study is available from the corresponding author upon reasonable request.
